# Factors associated with one-year mortality after hip fracture in people older than 85 years in Northern Sweden

**DOI:** 10.1007/s41999-025-01317-6

**Published:** 2025-10-06

**Authors:** Erika Olofsson, Yngve Gustafson, Sebastian Mukka, Laura Corneliusson, Eva Tengman, Lenita Lindgren, Birgitta Olofsson

**Affiliations:** 1 Department of Nursing, Umeå University, Umeå, Sweden; 2 Department of Community Medicine and Rehabilitation, Geriatric Medicine Division, Umeå University, Umeå, Sweden; 3 Department of Diagnostics and Intervention, Umeå University, Umeå, Sweden; 4 Department of Community Medicine and Rehabilitation, Physiotherapy Division, Umeå University, Umeå, Sweden

**Keywords:** Mortality, Hip fracture, Older adults, Northern Sweden

## Abstract

**Aim:**

To explore factors associated with one-year mortality risk after hip fracture among very old adults (85 +) in Northern Sweden.

**Methods:**

This cohort study includes data derived from Umeå 85 + /Gerontological Regional Database (GERDA), encompassing a representative sample of those aged 85, 90 and ≥ 95 years in Northern Sweden. Data were assessed during home visits and interviews conducted between 2000 and 2017. Associations of baseline characteristics with hip fracture during a follow-up period of 5 years and one-year mortality were analyzed using Cox proportional hazards regression.

**Results:**

Of 1,277 participants, 184 (14.4%) sustained a hip fracture during follow-up, of whom 47.8% died within 1 year of the fracture. Among those with hip fracture, 65% were women. Increased 1-year mortality risk was associated with the presence of depressive disorders (hazard ratio, HR 2.55, 95% confidence interval 95%CI, 1.32–4.93), history of stroke (HR 2.34, 95%CI 1.17–4.66) and subtrochanteric fractures (HR 4.40, 95%CI 1.73–11.21). Conversely, obesity (HR 0.26, 95%CI 0.10–0.67) was associated with reduced mortality risk.

**Conclusion:**

Nearly half of all very old adults sustaining a hip fracture die within 1 year. Depressive disorders, history of stroke and subtrochanteric fractures were identified as significant predictors of increased mortality, whereas obesity appeared to mitigate mortality risk. These findings underscore the need for targeted interventions to manage these risk factors in hip fracture patients very old adults.

## Key summary points

**Aim:**

To explore factors associated with one-year mortality risk after hip fracture among very old adults (85 +) in Northern Sweden.

**Findings:**

Nearly half of all very old adults sustaining a hip fracture die within 1 year**,** where those with depressive disorders, history of stroke and subtrochanteric fractures was factors associated increased risk while obesity decreased the mortality risk.

**Message:**

The mortality is overall high among very old adults with hip fracture, especially in certain subtypes, highlighting the need for improved fall prevention and post-hip fracture interventions with a focus on the most frail and oldest subgroups.

## Introduction

Falls among older adults frequently result in hip fractures, a serious complication with a persistently high mortality rate, despite improvements in surgical and anesthetic methods [1–3]. Previous studies suggest that between 20 and 25% of individuals die within the first year after a hip fracture [4–6]. Sweden experiences an annual incidence of roughly 16,000 hip fractures, the average age at the time of fracture being 80 years [7]. The risk of post-hip fracture mortality increases exponentially with advancing age [6, 8]; previous research has identified several contributing factors to the increased mortality, including age [9], sex [2, 9], fracture type [4] and dementia [10, 11]. However, there is a notable gap in the extant literature concerning factors associated with 1-year mortality among very old adults (85 +).

Although it is well established that hip fracture incidence increases with age, few studies have specifically examined this risk with a sample of very old adults. Most existing studies involving hip fracture patients report a mean age of 80 years [7], yet the most vulnerable population is likely those aged ≥ 85. This study aimed to explore factors associated with one-year mortality risk after hip fracture among very old adults (85 +) in Northern Sweden.

## Methods

### Study design

This study uses data from the Umeå 85+/Gerontological Regional Database (GERDA) study, which is a population-based investigation conducted by Umeå University, Sweden, across multiple periods: 2000–2002, 2005–2007, 2010–2012 and 2015–2017. The study adhered to the principles outlined in the Declaration of Helsinki and received ethical approval from the Swedish Ethical Review Authority (Dnr 99-326, 05-063 M, 2021-00023).

### Participants and settings

Participants were selected from the National Tax Agency registers, including every other individual aged 85 years (from a randomized starting point), all individuals aged 90 years and all those aged ≥ 95 years living in one urban and five rural municipalities within Västerbotten County. All eligible participants received study information and a formal invitation to participate. Subsequently, informed consent was obtained through telephone calls, and for those who consented, home visits were scheduled. For participants with cognitive impairment, consent was obtained from relatives or legal guardians.

**Fig. 1:**
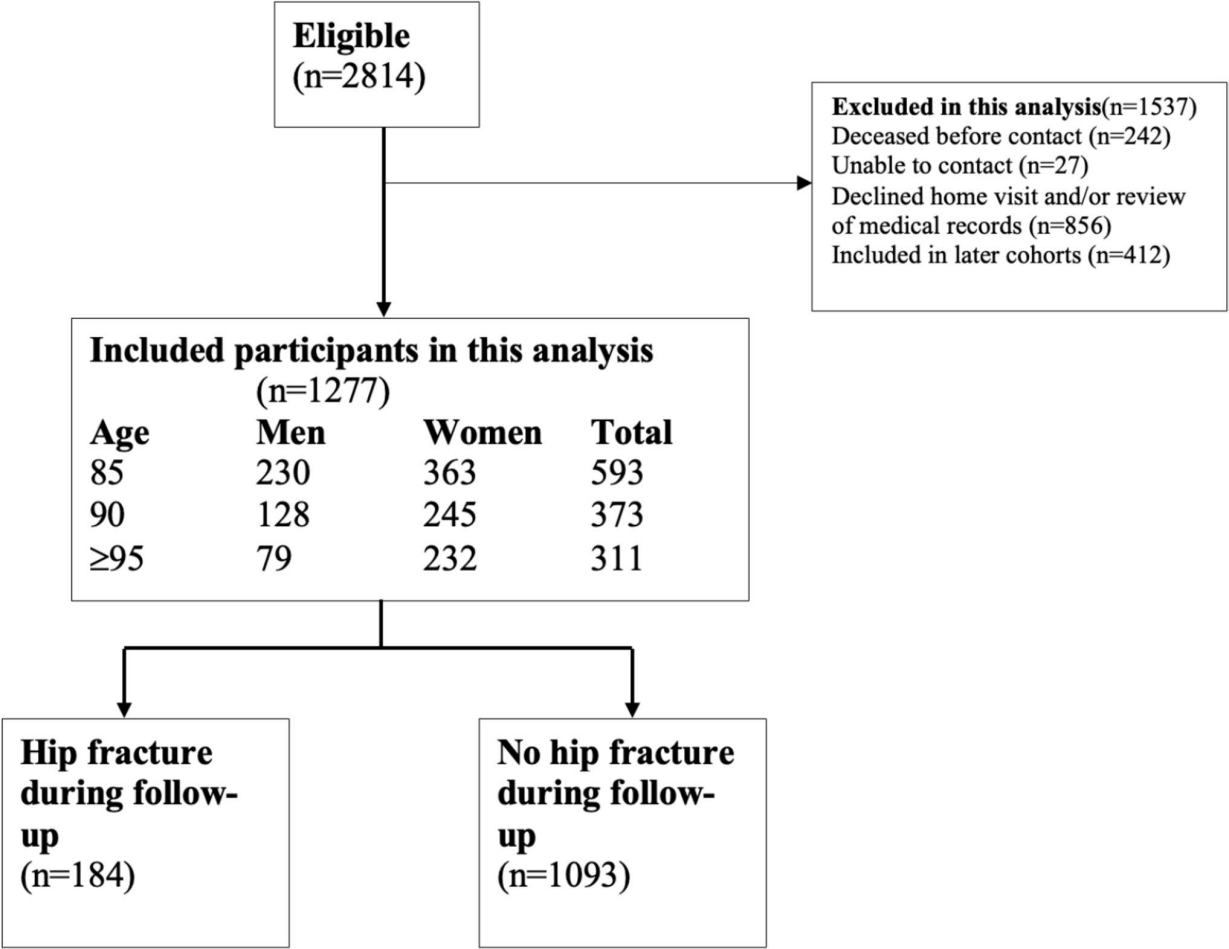
Flowchart of included and excluded participants in this analysis

Of the 2,814 individuals invited to participate between 2000 and 2017, the present study includes analyses of 1,277 participants from their earliest occasions of participation, all of whom had completed both home visits and medical record reviews (Fig. 1).

Trained assessors (nurses, physicians, medical students and physiotherapists) with prior medical knowledge conducted interviews and assessments inspired by the Comprehensive Geriatric Assessment (CGA) process during the home visits. The home visits were carried out at the participants’ residences, either in their homes or residential care facilities. Additional information was collected from care personnel and/or relatives acting as proxy respondents for participants residing in residential care facilities or when cognitive impairment necessitated such support. Medical records were reviewed to verify diagnoses and medications. For participants involved in multiple data collection periods, data from the earliest occasion, including home visits and medical record reviews, were used in the current analyses.

### Data and baseline variables

Data on the occurrence and classification of hip fractures between January 1, 1980 and February 1, 2023 were obtained through a comprehensive review of medical records and discharge registers from three local hospitals—Umeå, Skellefteå and Lycksele—managed by the County Council of Västerbotten. Hip fractures were classified into three types: femoral neck, trochanteric and subtrochanteric.

The follow-up period commenced on the date of study inclusion and concluded upon the first occurrence of either a hip fracture or death within a 5-year follow-up period post-inclusion. Dates of death were obtained from death certificates, electronic medical records and population registers.

Baseline variables were selected based on previous research examining the associations between falls, hip fractures and osteoporosis in older adults.

Cognitive function was assessed using the Mini-Mental State Examination (MMSE), where scores range from 0 to 30; a score of ≤ 23 signifies impaired cognition [12]. Depressive disorders were screened for using the 15-item Geriatric Depression Scale (GDS-15), a yes/no questionnaire with a scoring range of 0–15. The interpretation of GDS-15 scores classifies individuals into three categories: normal (0–4), experiencing mild depression (5–9) or suffering from moderate-to-severe depression (10–15) [13]. The GDS-15 has demonstrated high sensitivity and specificity for detecting clinical depression in individuals aged ≥ 85 [14], including those residing in residential care facilities [15] and older adults with mild or moderate cognitive impairment [16]. In addition to the GDS-15, the Life Orientation Scale and the Organic Brain Syndrome Scale were used to assess depressive status. The Philadelphia Geriatric Center Morale Scale (PGCMS) is a 17-item scale employing a 0–1 scoring system; higher/lower total scores reflect morale and subjective well-being [17, 18].

Body weight and height were measured to calculate body mass index (BMI, kg/m^2^), which was subsequently categorized as underweight (BMI < 22), normal weight (BMI 22–29) and high (overweight or obesity; BMI ≥ 30) [19]. Nutritional status was evaluated using the Mini-Nutritional Assessment (MNA) Scale, with scores ranging from 0 to 30. Malnutrition is indicated by scores < 17; scores between 17 and 23.5 indicate a risk of malnutrition; and scores ≥ 24 reflect good nutritional status [20].

Residents in care facilities with around-the-clock access to staff and nursing support were classified as living in a residential care facility. The 10-item Modified Barthel Activities of Daily Living (ADL) index, with scores ranging from 0 to 20, was used to assess dependence on personal activities of daily living (P-ADL) [21], where a score of 20 represents total independence.

Socio-demographic information was collected through interviews, while medical history and current medication use were subsequently verified by reviewing medical records. A consultant geriatrician (YG) confirmed pre-existing diagnoses of osteoporosis, primarily based on low-energy fractures and/or dual-energy X-ray absorptiometry (DXA) assessments. The geriatrician also verified diagnoses of dementia and depressive disorders by analyzing all available medical records, including current medical treatment and baseline assessments, which comprised the MMSE, GDS-15, Modified Barthel ADL, the Organic Brain Syndrome Scale (OBS-Scale), the Life Orientation Scale and the PGCMS. This comprehensive review determined whether participants met the DSM-IV-TR criteria for dementia or depressive disorders. The full diagnostic procedure has previously been described [22, 23].

### Statistical analysis

Categorical and continuous variables were compared between participants who died and those who survived 1-year post-hip fracture. Baseline characteristics were selected based on previously known risk factors and clinical relevance. In the appendix, the percentages are calculated in columns, whereas in Table 1, percentages are presented in rows.

**Table 1:**
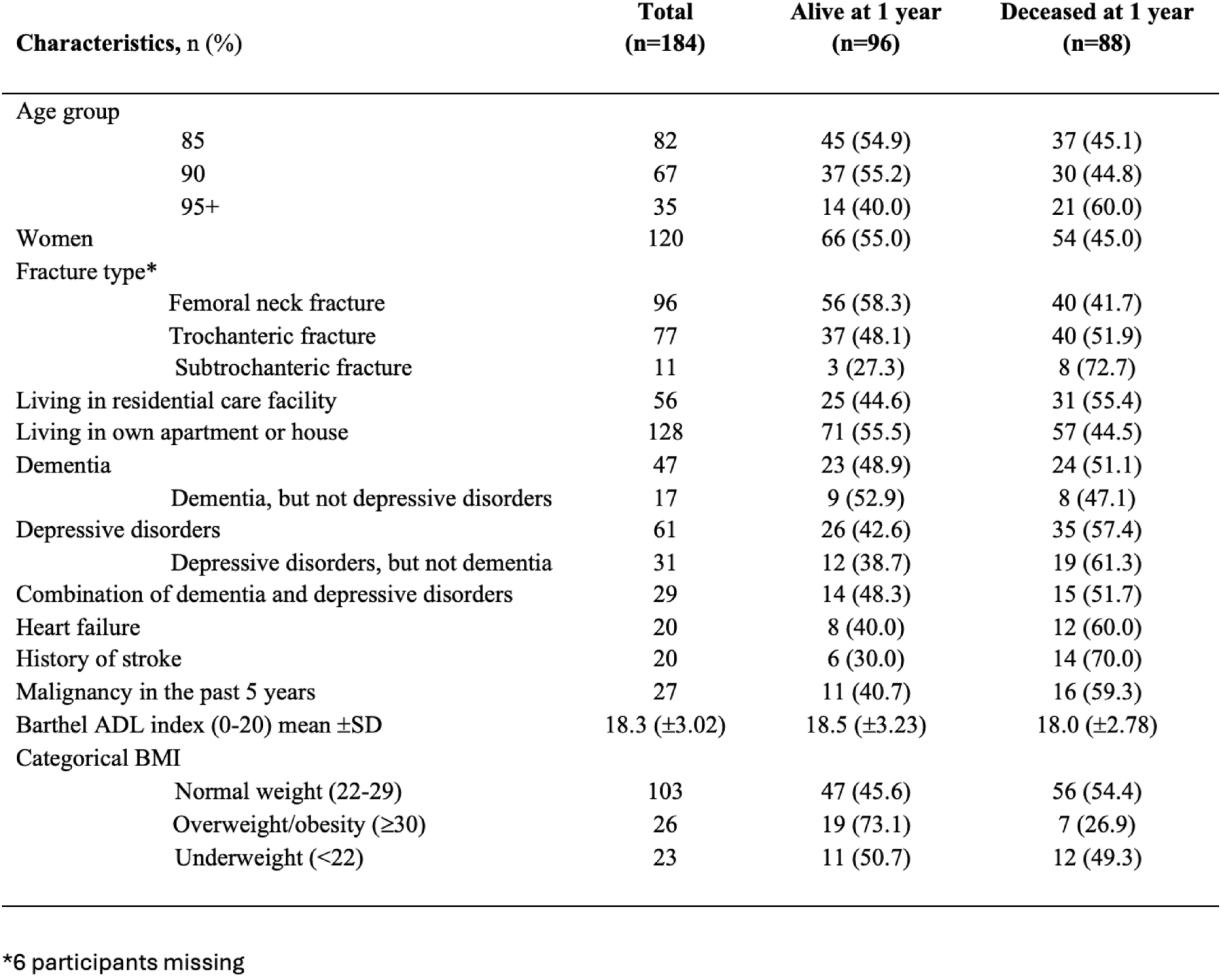
Baseline characteristics of participants by survival status 1 year after hip fracture

*ADL* Activity of Daily Living, *BMI* Body Mass Index, *SD* Standard Deviation

Time to hip fracture was calculated from the date of baseline assessments to the occurrence of a hip fracture within a 5-year follow-up period for those who experienced a fracture or until the date of the last follow-up for those who did not sustain a fracture.

Survival time was defined as the interval from the date of hip fracture (during follow-up) to the date of death (for participants who died during the follow-up) or to the date of the last follow-up date, 1 year after hip fracture.

Cox proportional hazard regression was used to identify mortality risk factors after hip fracture, with results presented as hazard ratios (HRs) and their corresponding 95% confidence intervals (CIs). A multivariable model, adjusted for previously known factors, including age group, fracture type, sex, residence in a residential care facility, cognition, heart failure, stroke, previous activity in daily life and BMI, was included in the analysis as covariates.

The proportional hazards assumption was evaluated by visually inspecting graphs based on weighted Schoenfeld residuals. Pearson and Spearman correlation coefficients were calculated for all variables; no correlations exceeding r = 0.6 were observed. A two-sided p-value of < 0.05 was considered statistically significant. Statistical analyses were conducted using IBM SPSS software (version 28).

## Results

### Sample characteristics

A total of 1,277 participants in Northern Sweden were included in this analysis of very old adults. Most of very old adults resided independently in their homes (64.3%) and were independent in transferring (84.7%), with the assistance of a walking aid (76.4%). Almost one-third of all adults over 85 years had dementia (34.8%) or a depressive disorder (35.1%). During follow-up, 184 participants (14.4%) sustained a hip fracture, and 239 (18.7%) had previously experienced a hip fracture before baseline (Appendix 1).

Of the very old adults who sustained a hip fracture during follow-up (n = 184), a substantial majority (65.2%) were women, and a large proportion resided independently before the fracture event. Moreover, 68.5% used walking aids before fracture, compared to 77.7% among those who did not sustain a hip fracture (Appendix 1).

Of the participants who sustained a hip fracture during follow-up, 33.2% (61 individuals) had depressive disorders and 25.5% (47 individuals) presented with dementia. Moreover, 29 (15.8%) participants had both dementia and depressive disorders. The most common fracture type was a femoral neck fracture (52.2%). The majority (67.8%) exhibited normal weight (BMI 22–29) (Table 1).

### Mortality

At 1-year post-hip fracture, almost half (47.8%) of the very old adults who sustained a hip fracture during follow-up had died, with a median survival of 61 days (IQR 149) (Table 1). Women had a median survival of 61 days (IQR 138), while men had a median survival of 48 days (IQR 162).

The highest mortality risk was observed in the oldest age group (95 + years), with a median survival of 42 days (IQR 61). The 90-year-old cohort had a median survival of 47 days (IQR 144), whereas the 85-year-old group demonstrated the longest median survival at 97 days (IQR 190).

Among individuals living in residential care facilities, 31 (55.4%) had died within the first year, in contrast to 57 (44.5%) of those living independently at home. Moreover, a higher proportion of adults over 85 years with pre-existing conditions, such as depressive disorders (57.4%), stroke (70.0%) and heart failure (60.0%), succumbed within the first year after hip fracture (Table 1.)

In the multiple Cox regression model, having a subtrochanteric fracture (HR: 4.40, 95% CI: 1.73–11.21), depressive disorders without dementia (HR: 2.55, 95% CI: 1.32–4.93) and a history of stroke (HR: 2.34, 95% CI: 1.17–4.66) were independently associated with an increased mortality risk within 1 year after hip fracture. In contrast, pre-fracture overweight or obesity was associated with reduced 1-year mortality risk (HR: 0.26, 95% CI: 0.10–0.67) (Table 2).

**Table 2:**
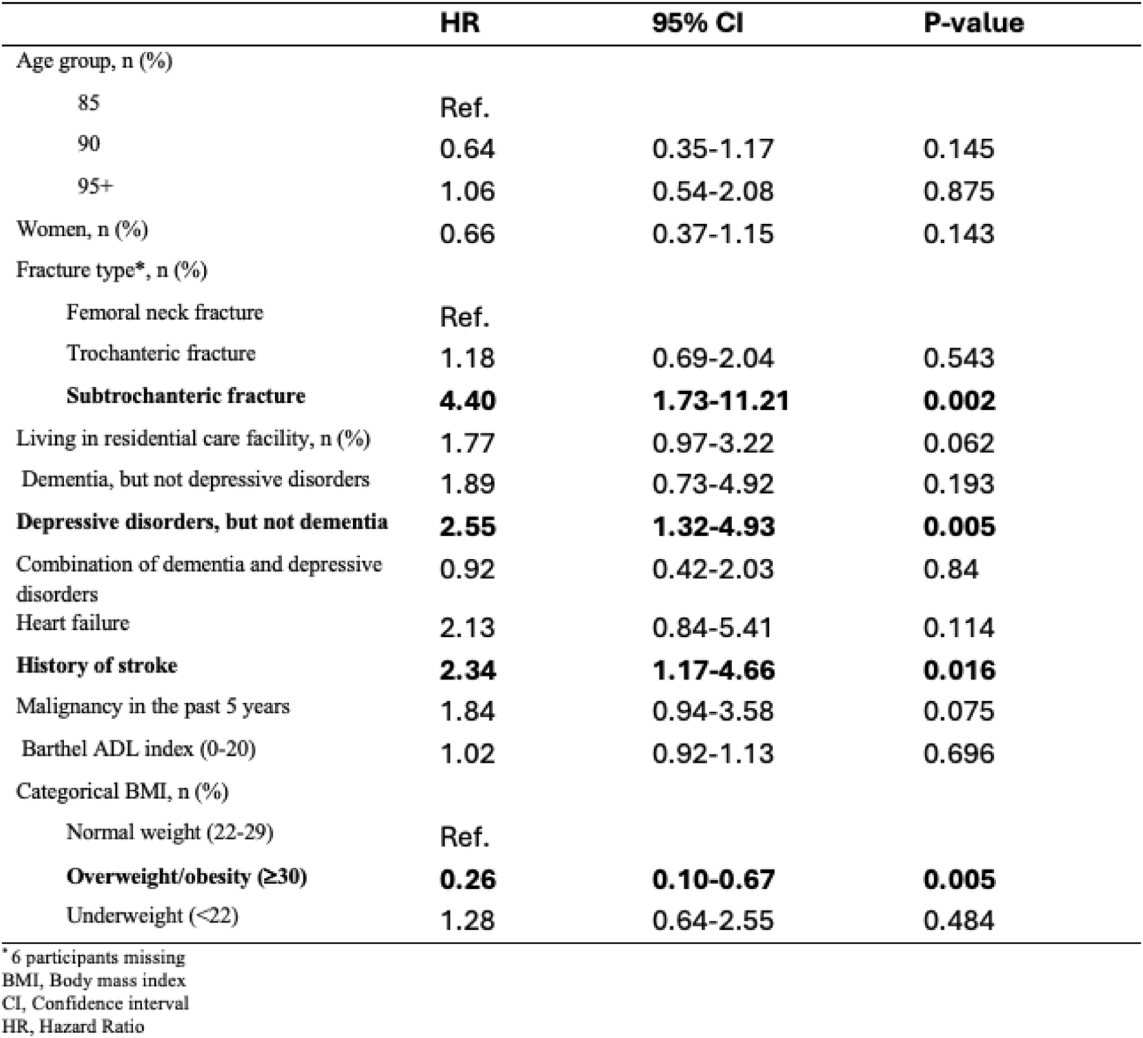
Cox proportional hazard model to assess 1-year mortality risk after hip fracture in the total cohort

**Fig. 2:**
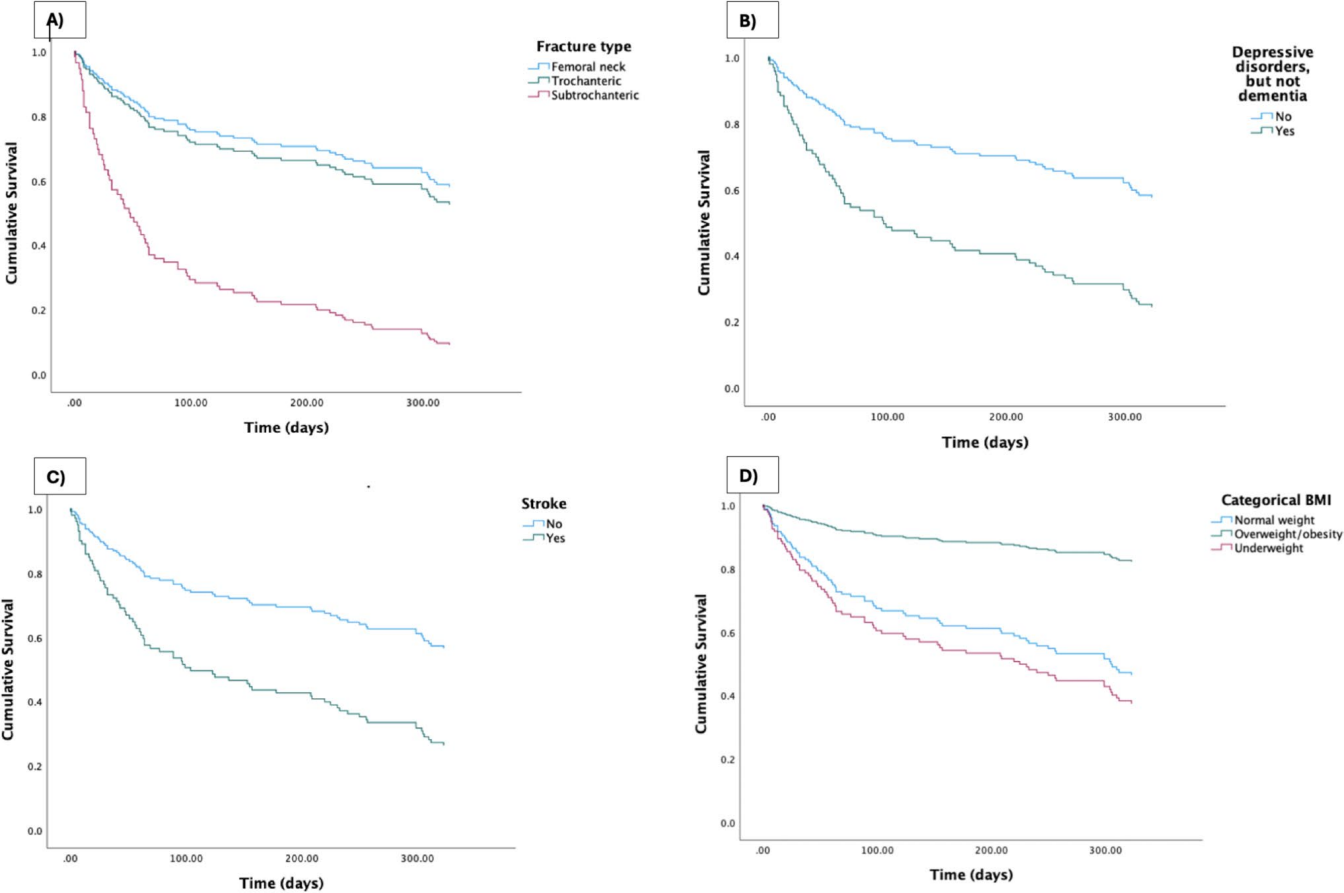
Survival function was estimated using the Kaplan–Meier method, with 1-year mortality as the endpoint. Data are stratified by significant variables identified in the Cox proportional hazard model (Table 2): **A** fracture type, **B** depressive disorders without dementia, **C** history of stroke before hip fracture and **D** body mass index (BMI)

To further visualize the variables that were significant in the multiple Cox model, a Kaplan–Meier analysis was performed, stratified by fracture type (Fig. 2A), history of depressive disorders without dementia (Fig. 2B), history of stroke (Fig. 2C) and BMI (Fig. 2D).

## Discussion

This study highlights the impact of hip fractures among very old adults in Northern Sweden. The findings confirm that mortality rates remain high following a hip fracture, particularly among those with pre-existing conditions such as depressive disorders or stroke and among those who sustain a subtrochanteric fracture. In contrast, obesity appears to improve survival among very old adults with a hip fracture.

Existing studies indicate that among patients with a mean age of 83, over 20% of female and > 30% of male patients die within 1 year after their hip fracture [2, 24]. This study of very old adults revealed a markedly elevated mortality rate, with nearly 50% of patients dying within the first year. This increased risk may be attributed to greater frailty and a higher prevalence of comorbidities in this population. These findings align with the literature, demonstrating a twofold increase in mortality risk in individuals over 90 years of age following hip fracture compared to younger adults [25]. A notably high mortality rate was observed in adults over 85 years with subtrochanteric fractures, with over 70% dying within the first year. In comparison, a study using data from the Swedish Fracture Register, which included 2,288 individuals with subtrochanteric fractures and a mean age of 81.0 years, reported a 1-year mortality rate of 24% [8].

Depressive disorders affect 20–32% of the very old adults and 32–65% of those with dementia. Additionally, factors such as dementia and reduced ADL capacity are associated with depressive disorders [23]. In this study, very old adults with a depressive disorder before hip fracture had a significantly increased mortality risk compared to older adults without depressive disorder, a finding supported by other studies on hip fracture patients [10, 26]. Other research suggests that depressive disorders in very old adults are underdiagnosed and undertreated, with fewer than half receiving antidepressant medications [23, 27]. Moreover, these studies underscore the need for further research into both antidepressant treatments and non-pharmacological interventions for depressive disorders in very old adults.

This study revealed a significantly lower mortality rate among very old adults classified as overweight or obese. This phenomenon, referred to as “the obesity paradox,” has been reported in previous research, although its underlying pathophysiological mechanisms remain poorly understood. Specifically, studies on patients with hip fractures have demonstrated that being overweight or obese is associated with a lower 1-year mortality risk compared to individuals with normal or underweight [28, 29], a trend also observed in adults over 85 years without hip fractures [30, 31]. These findings suggest that greater attention should be paid to malnourished older adults.

Findings from this study underscore the need for targeted interventions to manage these risk factors in hip fracture patients over 85 years. Proactive implementation of fall prevention measures in older adults is crucial for reducing hip fracture occurrence. Depressive disorders are not a normal part of ageing, highlighting the need for screening and optimizing treatment for depression in this population. Additional research is necessary to evaluate the effectiveness of post-hip fracture interventions in enhancing outcomes for older adults, with a focus on the most frail and oldest subgroups.

This population-based study included representative samples from all age groups across all cohorts, maintaining consistent sample selection and inclusion criteria throughout each data collection period. Another strength of this study is the use of in-home data collection, which allowed for the inclusion of participants who might have been otherwise excluded due to cognitive impairment or inability to travel. Inspired by CGA, assessments yielded superior data quality in-home visits, surpassing questionnaires or registry data. Before each new data collection, assessors underwent training to ensure assessment quality. A standardized diagnostic data collection procedure was used, and an experienced geriatrician (YG) consistently evaluated the data against DSM-IV criteria, thereby ensuring diagnostic quality and consistency. However, the study’s generalizability may be limited due to its exclusive focus on a single region in Sweden, with different access to orthogeriatric care. Additionally, a non-participation rate of approximately 28% among eligible patients may have compromised the generalizability of the findings. The extended follow-up duration from baseline assessment might have led to the non-detection of changes in medical conditions, diseases, drug prescriptions and functional capacity. Considering the study group's high age and potential frailty, a shorter follow-up period might have been more appropriate.

## Conclusion

Nearly half of very old adults who sustain a hip fracture die within one year. Depressive disorders, a history of stroke, and subtrochanteric fractures were identified as significant predictors of increased mortality. In addition, the “obesity paradox” appears to apply to this very old population with hip fractures.

## Data Availability

The datasets used and/or analysed during the current study are available from the corresponding author on reasonable request.
